# Endogenous Endopthalmitis in Disseminated Methicillin-Sensitive Staphylococcus aureus (MSSA) Bacteremia

**DOI:** 10.7759/cureus.34707

**Published:** 2023-02-06

**Authors:** Noor Amalina Saidi, Qi Zhe Ngoo, Shawarinin Jusoh, Muhammad Fadhli Ab Hamid, Wan Norliza Wan Muda

**Affiliations:** 1 Ophthalmology and Visual Science, School of Medicine Sciences, Kelantan, MYS; 2 Ophthalmology, School of Medical Sciences, Universiti Sains Malaysia, Kota Bharu, MYS; 3 Ophthalmology, Hospital Tengku Ampuan Afzan, Kuantan, MYS; 4 Ophthalmology, Hospital Kuala Lipis, Kuala Lipis, MYS

**Keywords:** uncontrolled diabetes, immunosuppressed, metastatic, mssa bacteraemia, endogenous endophthalmitis

## Abstract

Endogenous endophthalmitis (EE) is an ocular infection resulting from hematogenous spread from the remote primary source. Risk factors include endocarditis, bacteria meningitis, immunosuppressive state, and invasive procedures in patients with sepsis. We present a case of a 43-year-old gentleman with poorly controlled diabetes mellitus who was admitted for bilateral nasoseptal cellulitis with a right nasal wall abscess and right vocal cord palsy. At presentation, he just had preseptal cellulitis without any posterior segment involvement. He underwent incision and drainage under the Otorhinolaryngology team. Unfortunately, postoperatively he developed sepsis with a hematogenous spread of infection systemically involving his right eye (endophthalmitis) and his heart valve (infective endocarditis). Blood culture revealed Methicillin Sensitive Staphylococcus Aureus (MSSA) infection. He had six weeks of intravenous cloxacillin and three times intravitreal injections of vancomycin and ceftazidime with complete resolution of signs and symptoms. In the case of a poorly controlled diabetic patient with an extensive regional infection, the presence of ocular symptoms and signs that are suggestive of EE must be taken seriously and warrant a complete eye examination as early detection and treatment of EE is crucial for better prognosis.

## Introduction

Endogenous endophthalmitis (EE) is one of the ophthalmic emergencies as it can lead to devastating sight-threatening complications. It is an ocular infection that results from the metastatic spread of infection from a remote primary source [1,2]. This is a case report of extensive nasoseptal cellulitis with a right nasal wall abscess that had spread hematogenously during incision and drainage procedure and became methicillin-sensitive Staphylococcus aureus (MSSA) sepsis with endophthalmitis and infective endocarditis in a structurally normal heart valve.

## Case presentation

**Figure 1:**
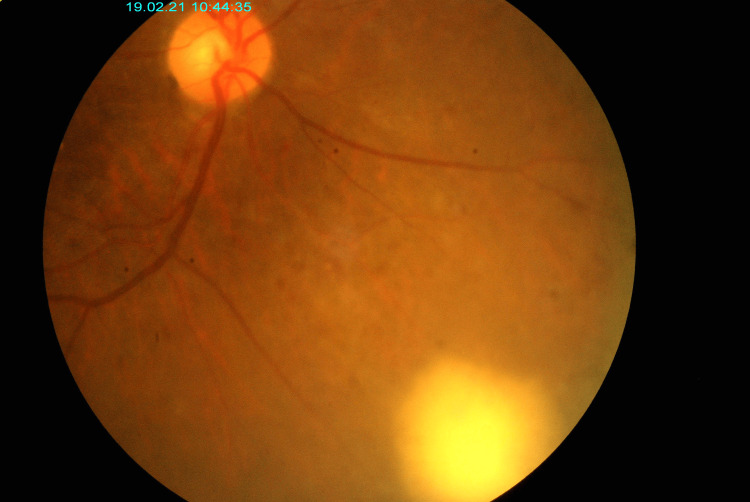
Fundus photo of the right eye with slightly hazy media with vitritis and large focal retinitis at inferonasal retina

A 43-year-old gentleman with poorly controlled diabetes mellitus was admitted for bilateral nasoseptal cellulitis with a right nasal wall abscess that has spread to the nasopharynx causing right vocal cord palsy. At presentation, he had a feature of preseptal cellulitis without any evidence of posterior segment involvement of infection. He underwent incision and drainage by the Otorhinolaryngology team. Postoperatively he deteriorated into sepsis and complaint of worsening vision, especially over the right eye. Upon examination, relative afferent pupillary defect (RAPD) was negative, vision right eye (OD) was counting finger, and left eye (OS) 6/60. RE showed injected conjunctiva with minimal chemosis, the cornea was clear with anterior chamber cells and flare. There were anterior vitreous cells, and the fundus was slightly hazy due to vitritis with unifocal retinitis at the inferonasal retina (Figure 1). B scan showed retina flat with loculations. LE anterior segment was normal, and the fundus showed moderate to severe non-proliferative diabetic retinopathy with centrally involved macula edema that explained the poor vision over the LE. Intraocular pressure of both eyes was normal.

**Figure 2:**
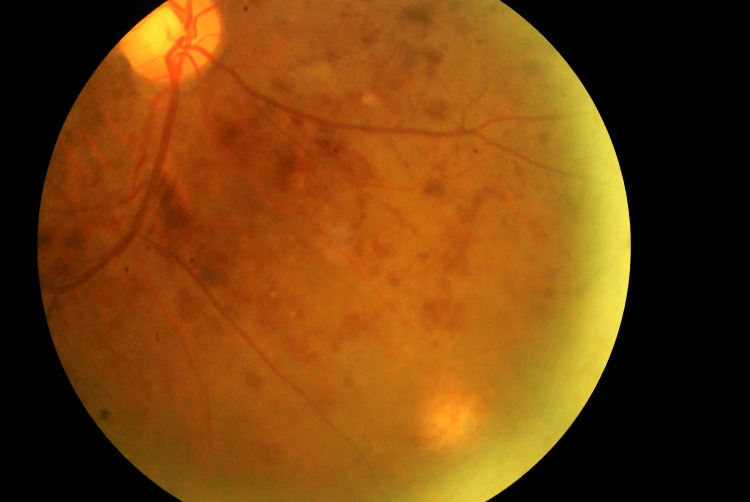
Fundus photo of the right eye after three months of presentation with resolution of vitritis and contracting focal retinitis inferonasal retina

Other than ocular spread, dissemination of infection also affected other organs leading to infective endocarditis, septic emboli to the lungs, and also to the right internal jugular vein causing thrombosis. Other investigations as viral markers and malignancy screening were taken to rule out other sources of immunosuppression than uncontrolled diabetes was negative, and the patient was not taking any immunomodulating drugs. His Blood culture revealed MSSA infection however his vitreous fluid culture was negative. He was then treated as RE endogenous endophthalmitis secondary to disseminated MSSA bacteremia and received multiple intravitreal injections of vancomycin and ceftazidime. He responded well with a course of intravenous cloxacillin for six weeks. Similarly, his eye responded well to treatment with resolution of vitritis and retinitis in RE (Figure 2). The nasoseptal abscess and endocarditis also resolved with the completion of the antibiotics. The patient was started on an anticoagulant for three months and subsequently was discontinued after the internal jugular vein thrombosis had cleared. However, he subsequently developed left ischemic central retina vein occlusion that required laser treatment and the visual recovery of his right eye was poor with improvement from counting finger to 6/60. While his left eye underwent grid laser for diabetic macula edema and showed improvement during follow-up with vision of 6/18 unaided and 6/9 with pinhole.

## Discussion

Endogenous endophthalmitis (EE) accounts for 2%-8% of endophthalmitis cases [2,3]. It is an ocular infection resulting from hematogenous spread from a remote primary source, in which the most common foci are from the liver abscess, followed by pneumonia, endocarditis, and soft tissue infection. EE is often associated with underlying risk factors including immunosuppressive state, uncontrolled diabetes mellitus, intravenous drug abuse, indwelling catheters, and invasive procedures, especially in sepsis patients [4].

The causative organisms for endogenous endophthalmitis may vary from a wide range of bacteria or fungal agents and it differs geographically. Gram-positive organisms such as Staphylococcus and Streptococcus are particularly more common in developed countries, while gram-negative organisms predominate in Asian populations [3,5], and liver abscess due to Klebsiella pneumoniae is the main source of EE [6]. While Zhang et al. reported that the commonest organisms found in EE were fungal about 60% followed by gram-positive and gram-negative bacteria [7]. In EE, fungal positive culture usually reveals Candida in comparison to exogenous endophthalmitis whereby Aspergillus and Fusarium are typically present.

Patients with EE may be presented with sudden onset of reduced vision associated with eye redness, eyelid swelling, eye pain, photophobia, and floaters [8]. An ocular examination may show conjunctiva injection, chemosis, the presence of cells, and flares with fibrin and hypopyon in the anterior chamber. Posterior segments often show vitritis with or without chorioretinitis. Gradual onset of symptoms with a characteristic string of pearls vitreous opacity and chorioretinitis are suggestive of fungal etiology. Without treatment, the disease may progress to pan ophthalmitis, cornea perforation, and end up with phthisis bulbi [9].

In septicemia, as we report in this case with MSSA sepsis, the treating physicians must be aware of the possibility of endogenous spread of infection to the eye, especially if associated with sudden onset of reduced vision during the course of illness together with other classic ocular features of EE [2,10].

In establishing the diagnosis of EE, an investigation tool such as a B scan is helpful especially in hazy ocular media to identify vitritis and vitreous exudates that will be visible as vitreous locations. B scan can also demonstrate the presence of other complications such as retina or choroidal detachment. Systemic workup for underlying systemic infection is necessary to identify the source of infection. Blood culture can demonstrate the presence of the systemic causative agent. However, in culture-negative cases, polymerase chain reaction (PCR) of either aqueous or vitreous samples is better in identifications of both bacteria and fungi [2,10]. Vitreous sampling and cultures are essential in all cases of EE to identify the causative organism and for correct targeted local and systemic treatment [10]. Vitreous sampling can be obtained by either needle aspiration of vitreous tap or vitrectomy. Vitreous fluid sampling via vitrectomy yields better results compared to needle aspiration [11].

Management of EE involves both local and systemic treatment which is equally important. The local intravitreal antibiotic injection is important within 24 hours of diagnosis for a favorable outcome to allow delivery of adequate concentration of antibiotics at the target site [12]. Prompt treatment must be initiated even though the culture and sensitivity results are still pending. Kabbara et al. reported that the most common empirical intravitreal antibiotic injection includes Vancomycin for Gram-Positive coverage and Ceftazidime for Gram Negative [13]. Meanwhile, systemic antibiotics are equally important to treat the underlying source of bacteremia. Although the delivery of systemic antibiotics for ocular infection is limited by blood ocular barrier, the inflammation may make the barrier more permeable for better drug penetration [6,10,14].

Findings from the study by Koul et al. [15] in 1989 show administration of topical and systemic steroids gave better results than no steroids or only topical steroids for endophthalmitis. Robbins et al. [16] also concluded that Systemic corticosteroid therapy improved the visual outcomes in endophthalmitis, especially in patients that show evidence of severe inflammation as fibrinous reaction and conjunctival hyperemia. However, treatment with an oral corticosteroid to control the inflammation in case of EE might carry the risk of exacerbating a systemic infection [16].

The guideline for the surgical management of EE as pars plana vitrectomy (PPV) is not well established as in early vitrectomy study (EVS) which is applied for exogenous post-operative endophthalmitis. However, PPV may aid in reducing and removing the nidus of intraocular infection [13]. At present, there are many studies and reports available that demonstrated good and favorable outcomes with a combination of systemic antibiotics with vitrectomy [17-20].

## Conclusions

EE often occurs secondary to underlying primary systemic pathology. It is an ophthalmological emergency as the visual outcome of EE can be devastating. A high index of suspicion of EE is very important, especially in patients with potential risk factors associated with eye signs and symptoms. Patients with uncontrolled diabetes are typically immunocompromised, which is often overlooked. Therefore, physicians should be alert to the early signs and symptoms of EE in this group of patients. Prompt diagnosis and management are crucial for better visual outcomes for patients with EE. Patients should be followed up closely for the response to the treatment and they must be explained the possible complications and visual prognosis of EE. A multidisciplinary approach by physicians and vitreoretinal surgeons is of utmost importance in the management of EE.
